# Comments on the “Spontaneous Coronary Reperfusion in STEMI After Pre‐Hospital Loading Dose of Triple Antithrombotic Therapy”

**DOI:** 10.1002/clc.70472

**Published:** 2026-09-22

**Authors:** Muhammad Atif, Hamed Khan, Iqra Alam, Hamad Ullah

**Affiliations:** ^1^ Saidu Medical College Saidu Group of Teaching Hospitals Swat Khyber Pakhtunkhwa Pakistan

Dear Editor,

Having read the recently published article by Meunier et al. paper [1]. This study evaluates spontaneous coronary reperfusion (SCR) in patients with ST‐segment elevation myocardial infarction (STEMI) treated with pre‐hospital triple antithrombotic therapy before primary percutaneous intervention PCI, we can state that the authors deserve appreciation for performing an impactful prospective bicentric study on the topic that is of significant importance, but remains understudied. The discovery of spontaneous TIMI 3 flow in almost one‐fifth of patients, along with the reduction in infarct‐related biomarker levels, provides novel and highly relevant information to the existing literature. However, some aspects need to be highlighted since they might affect the interpretation of the main findings of the study.

To begin with, there was heterogeneity of the antithrombotic therapy applied to the study sample despite the fact that it was considered as a group receiving a certain treatment. All the patients were pre‐treated with aspirin and anticoagulation before hospital admission, but different P2Y12 inhibitors were used; ticagrelor, clopidogrel, prasugrel, and cangrelor, while two types of anticoagulant therapy were utilized; enoxaparin and unfractionated heparin. The main thing is that the clopidogrel was preferred for patients with contraindications to stronger P2Y12 inhibitors. This pharmacological heterogeneity might have mitigated treatment‐specific effects on spontaneous reperfusion, making it impossible to conclude that pre‐hospital P2Y12 loading had no effect on the development of spontaneous TIMI 3 flow. In recent pharmacodynamics studies, differences in antiplatelet efficacy, mode of delivery, and timing had a significant impact on the degree of platelet inhibition prior to primary PCI [2].

Second, while the time from symptom onset to PCI was provided in the study, no information about the time interval between loading‐dose administration and coronary angiography was included in the results. This time interval can be considered one of the key factors that determine the degree of pre‐procedural platelet inhibition, especially for oral P2Y12 inhibitors in the setting of acute STEMI, due to gastrointestinal absorption delay, the administration of opioids, and hemodynamic instability [3]. Hence, there could be insufficient pharmacodynamic effect rather than therapeutic equivalence in comparison of ticagrelor and clopidogrel, which would be more convincingly shown by reporting treatment‐to‐angiography time.

Lastly, the connection between spontaneous reperfusion and out‐of‐hospital cardiac arrest needs to be interpreted carefully. The study proposes that arrhythmias caused by spontaneous reperfusion may have contributed to the increased prevalence of cardiac arrest seen among patients with spontaneous TIMI 3 flow. However, as mentioned in the study, a coronary angiogram was performed only post‐hospital admission; therefore, making it impossible to determine whether spontaneous reperfusion took place before or after the arrhythmic episode or is just a coincidence. In the absence of such an established timeline, it would not be accurate to suggest causality in this observation [4].

We respectfully highlight the limitations of this study to better understand the outcomes in clinical practice.

## Author Contributions

Conceptualization: Iqra Alam, Muhammad Atif, Hamed Khan; Manuscript: Iqra Alam, Hamad Ullah. Data curation: Muhammad Atif, Iqra Alam. Investigation: Iqra Alam, Muhammad Atif; Review: Muhammad Atif, Hamed Khan; Supervision: Hamed Khan.

## Funding

The authors have nothing to report.

## Ethics Statement

Ethical approval was not required, as per standard practice and journal requirements.

## Conflicts of Interest

The authors declare no conflicts of interest.

## Data Availability

No new datasets generated. All the data have been taken from publicly available resources and can be accessed through the reference list.
